# Acute toxicity of chemotherapy in central nervous system germ cell tumour patients according to age

**DOI:** 10.3389/fonc.2024.1421418

**Published:** 2024-07-15

**Authors:** Gilles Palenzuela, Camille Schiffler, Didier Frappaz, Andreas Peyrl, Nicolas U. Gerber, Rolf-Dieter Kortmann, Michael Philippe, Martin Zimmermann, Matthew J. Murray, James C. Nicholson, Gabriele Calaminus, Cécile Faure-Conter

**Affiliations:** ^1^ University Hospital, Department of Paediatric Haematology and Oncology, Montpellier, France; ^2^ Department of Biostatistics, Centre Léon Bérard, Lyon, France; ^3^ Department of Pediatric Oncology, Institut d’Hémato‐oncologie Pédiatrique, Lyon, France; ^4^ Department of Paediatrics, Medical University of Vienna, Vienna, Austria; ^5^ Department of Oncology, University Children’s Hospital, Zürich, Switzerland; ^6^ Department of Radiotherapy, University Hospital, Leipzig, Germany; ^7^ Department of Clinical Pharmacy and Oncology, Centre Léon Bérard, Lyon, France; ^8^ Department of Paediatric Hematology and Oncology, Hannover Medical School, Hannover, Germany; ^9^ Department of Paediatric Haematology and Oncology, Cambridge University Hospitals, Cambridge, United Kingdom; ^10^ Department of Pathology, University of Cambridge, Cambridge, United Kingdom; ^11^ Department of Paediatric Haematology and Oncology, University Children's Hospital Bonn, Bonn, Germany

**Keywords:** germ cell tumour, chemotherapy, children, adults, adolescents, toxicity, age

## Abstract

**Background:**

SIOP-CNS-GCT-II European trial was opened for the treatment of patients of any age with central nervous system germ cell tumour (CNS-GCT). Four courses of pre-irradiation chemotherapy were delivered. The influence of patient age on chemotherapy related acute toxicity (CRAT) was assessed.

**Methods:**

CRAT was analysed according to age-groups: children (aged ≤11 years), adolescents (aged 12-17 years), adults (aged ≥18 years) and to chemotherapy type: CarboPEI (alternating etoposide-carboplatin/etoposide-ifosfamide) for non-metastatic germinoma; PEI (cisplatin-etoposide-ifosfamide) for standard-risk non-germinomatous GCT (NGGCT); PEI and high-dose PEI (HD-PEI), for high-risk or poorly responsive NGGCTs.

**Results:**

296 patients were assessable for CRAT: 105 children, 121 adolescents, 70 adults (max age: 41 years). Median cumulative doses/m² of chemotherapy were similar among age-groups. The proportion of germinoma over NGGCT (and accordingly use of CarboPEI chemotherapy) was higher in the adult groups (79%) versus the other two groups (62%). Delay in chemotherapy ≥7 days was noticed in 27%, 38%, and 19% of children, adolescents, and adults, respectively. Grade ≥3 haematological and non-haematological adverse events (AEs) were observed in 94%/31%, 97%/36%, and 77%/21% of children, adolescents, and adults, respectively. No toxic death was reported. Grade ≥3 AEs and delayed chemotherapies were significantly rarer in adults when compared with adolescents, even when adjusted on chemotherapy type: odds ratio: 0.1 [95%CI 0.02-0.4], and 0.2 [95%CI 0.1-0.4] in the group treated with CarboPEI.

**Conclusion:**

Adult patients can be treated safely with a chemotherapy intensive protocol, with even less toxicity than that observed in adolescents. Further work is required to understand age-related differences regarding toxicity.

## Introduction

CNS-GCT are observed in children, adolescents, and young adults (1). In the past, standard treatment was limited to radiotherapy, at least for pure germinoma (2). To avoid the toxicity of radiation on maturing brains, therapeutic strategies including pre-irradiation chemotherapies were developed by paediatric groups (3, 4).

IIn non-metastatic germinoma, the French TC 90 protocol consisted of two cycles of CarboPEI followed by radiotherapy to the primary (40 Gy) and laid the basis of the SIOP-CNS-GCT96 trial (5). Later on, in the SIOP-CNS-GCTII trial, 24 Gy whole ventricular irradiation was added to prevent ventricular relapses (6). In non germinomatous germ cell tumour (NGGCT), the European SIOP-CNS-GCT-96 trial, evaluated a strategy consisting of four courses of PEI (cisplatin-etoposide- ifosfamide), followed by 54 Gy focal radiotherapy in non-metastatic NGGCT (or 30 Gy craniospinal if metastatic) and identified a high-risk (HR) group of patients, namely those aged less than six years or with Alpha-fetoprotein (AFP) level in serum and/or cerebrospinal fluid (CSF) above 1000 ng/ml at diagnosis (7). In the subsequent SIOP-CNS-GCT-II European trial, whose acute toxicity is presented here, chemotherapy for the HR-NGGCT group was intensified with 2 courses of high doses (HD) of etoposide and ifosfamide (HD-PEI) following 2 courses of standard dose PEI. Meanwhile, the upper age limit in the trial was withdrawn.

The potential toxicity, and thus the feasibility of these more intensive chemotherapy protocols in adults, currently remain a matter of debate. Indeed, chemotherapy related acute toxicity (CRAT) might increase with patient age, as already observed in other tumours such as medulloblastoma (8). The objective of this study was therefore to compare the tolerance of chemotherapy across age-groups within the SIOP-CNS-GCT-II trial.

## Patients and methods

### Population

Records of patients included in the European SIOP-CNS-GCT-II protocol (EudraCT number 2009-018072-33) and treated with chemotherapy were analysed. The inclusion period spanned almost seven years (06/10/2011 to 01/07/2018). Patients were treated either in the pure germinoma or NGGCT group. Information regarding gender, patient age, general status, presence of diabetes insipidus (DI), site of primary tumour and type of tumour were collected at diagnosis.

### Treatment

**Figure 1:**
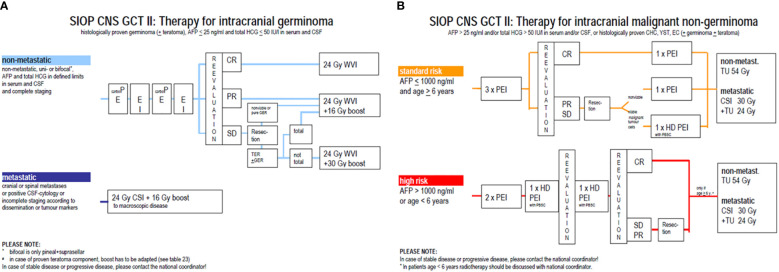
Overview of treatment for germinoma **(A)** and non germinomatous germ cell tumours **(B)**.

In the germinoma group (**Figure 1A**), only patients with localised tumours (no spinal nor intracranial metastasis and negative CSF cytology, including bifocal presentation in suprasellar and pineal areas) received chemotherapy: two cycles of CarboPEI (etoposide 100 mg/m² on days 1-3 and days 22-24, carboplatin 600 mg/m² on day 1, ifosfamide 1800 mg/m² on days 22-26), followed by 24 Gy whole ventricular radiotherapy and, in case of post chemotherapy residual disease, a 16 Gy boost on residual disease. Cumulative doses of chemotherapy in this group were as follows: etoposide 1200 mg/m², carboplatin 1200 mg/m², ifosfamide 18000 mg/m². Patients with either metastatic or incompletely staged germinoma received craniospinal radiotherapy alone and were not analysed in this series.

In the NGGCT group (**Figure 1B**), all patients, regardless of stage, received four courses of pre-irradiation chemotherapy. Patients with standard-risk (SR)-NGGCT [serum and CSF AFP ≤1000 ng/ml and age ≥6 years at diagnosis] received three courses of PEI (cisplatin 20 mg/m² on days 1-5, etoposide 100 mg/m² on days 1-3, ifosfamide 1500 mg/m² on days 1-5) every 21 days, followed by tumour imaging reassessment. In case of complete remission, a fourth course of PEI was administrated; otherwise, resection of any residual was recommended. If no viable malignant tumour was identified on pathological review of the resection specimen, a fourth course of PEI was administrated. Cumulative doses in this group were as follows: cisplatin 400 mg/m², etoposide 1200 mg/m², ifosfamide 30000 mg/m². If malignant viable tumour cells were present on pathology of the resection specimen, the fourth course consisted of HD-PEI (cisplatin 20 mg/m² on days 1-5, etoposide 300 mg/m² on days 1-5, ifosfamide 2000 mg/m² on days 1-5) with haematopoietic stem cell support on day 7. Cumulative doses of chemotherapy in this group were as follows: cisplatin 400 mg/m², etoposide 2400 mg/m², ifosfamide 32500 mg/m². Patients with HR-NGGCT (serum or CSF AFP >1000 ng/ml or age <6 years at diagnosis) received two PEI with harvest of hematopoietic stem cells and two HD-PEI before surgery of any resectable residual disease. Cumulative doses of chemotherapy in this group were as follows: cisplatin 400 mg/m², etoposide 3600 mg/m², ifosfamide 55000 mg/m². Then, all patients aged 6 years or more with SR and HR-NGGCT received 54 Gy focal irradiation to the initial tumour volume, with 30 Gy to the craniospinal axis for metastatic cases.

Data were analysed by therapeutic groups according to the three chemotherapy schemes: ‘CarboPEI’, ‘PEI’, and ‘PEI/HD-PEI’. Patients who received non-protocol chemotherapy or a combination of protocol chemotherapies not described above were excluded from the analysis.

### Chemotherapy-related acute toxicity

CRAT was assessed through two indicators: grade ≥3 adverse events (AE) and delayed chemotherapy. AE were recorded and graded prospectively in the database according to the common terminology criteria for adverse events (CTCAE) v4.0 scale. Grade ≥3 AE were analysed and split into haematological and non-haematological (peripheral neurotoxicity, infection/fever, gastrointestinal, renal, and auditory) AE.

Delayed chemotherapy was defined as an interval ≥28 days between two courses.

### Statistical analysis

Statistical analyses were performed using SAS (Statistical Analysis System) version 9.4 (SAS Institute). Categorical data were summarised by frequencies and percentages and presented by age-group. Logistic regression analyses were performed to assess whether patient age or therapeutic group was correlated with delayed chemotherapy and/or occurrence of grade ≥3 AE. The odds ratio (OR) was assessed with a confidence interval (CI) of 95%. P-values of <0.05 were considered statistically significant.

Written informed consent was obtained from all patients/guardians at diagnosis, and national ethics review boards approved the trial.

## Results

### Population and treatment

Among the 306 patients included in the SIOP-CNS-GCT-II trial, 10 were excluded: two patients did not receive chemotherapy, five received non-protocol chemotherapy, and three received a combination of CarboPEI and PEI. Among the 296 remaining patients (**Table 1**), 105 (35%) were children, 121 (41%) were adolescents, and 70 (24%) were adults. Patients were predominantly male (80%). Median age at diagnosis was 14 years (range 0 to 41 years). The primary tumour was pineal site in 52% of patients, suprasellar in 25%, bifocal (pineal and suprasellar) in 20%.

**Table 1:** Characteristics of patients and tumours at diagnostic by age groups.

	Children (0-11 y) n=105		Adolescents (12-17 y) n=121		Adults (≥18 y) n=70		All n=296	
Sex								
Male (%)	63	60%	108	89%	67	96%	238	80%
Female (%)	42	40%	13	11%	3	4%	58	20%
Age (years)								
N (missing)	105	0	121	0	70	0	296	0
Mean (std)	9	1,9	15	2	23	4,6	15	6
Median (min; max)	10	0;12	15	12; 18	22	18; 41	14	0; 41
General condition (Lansky/Karnofsky index)								
10-50 (%)	6	6%	8	7%	3	4%	17	6%
60-70 (%)	12	11%	12	10%	10	14%	34	12%
80-100 (%)	53	50%	77	64%	33	47%	163	55%
Not available (%)	34	32%	24	20%	24	34%	82	28%
Diabetes insipidus								
No (%)	52	50%	77	64%	48	69%	177	60%
Yes (%)	43	41%	42	35%	19	27%	104	35%
Not available (%)	10	10%	2	2%	3	4%	15	5%
Site of primary tumour								
Pineal (%)	47	45%	70	58%	38	54%	155	52%
Suprasellar (%)	42	40%	24	20%	9	13%	75	25%
Bifocal (pineal + suprasellar) (%)	15	14%	25	21%	19	27%	59	20%
Other site (%)	1	1%	2	2%	4	6%	7	2%
Diagnosis								
NGGCT (%)	41	39%	46	38%	15	21%	102	35%
Germinoma (%)	64	61%	75	62%	55	79%	194	66%

NGGCT, non-germinomatous germ cell tumour.

The majority (66%) of patients had germinoma, and this proportion was higher in adults (79%) than in children and adolescents (61 and 62%, respectively). All 194 patients with germinoma received CarboPEI. Among the 102 patients with NGGCT, 77 (76%) received PEI, and 24 (24%) received PEI/HD-PEI (eight received one HD-PEI and 16 received two). One child (1%) with a localised pineal tumour had a CSF HCG level (52 UI/L) just above the trial threshold but received CarboPEI and was analysed in germinoma group. Upon local physician’s choice, ten patients out of 296 (3%) had their chemotherapy doses reduced for all courses: for three adult patients, the body surface area (BSA) was capped at 2 m², leading to a 10% dose reduction for all the drugs (two patients in the CarboPEI group and one in the PEI group). For six adults and one adolescent (all in the CarboPEI group), carboplatin doses were capped at 1000 mg. In the PEI/HD-PEI therapeutic group, the mean doses of etoposide were 25% lower in the adult patients (2700 mg/m²) than in children and adolescents (3600 mg/m² and 3500 mg/m², respectively). Reasons for lowering the etoposide dosing in adults was not captured in the database; nevertheless, this represents the smallest therapeutic group with only four adult patients included. Despite those modifications, and with the exception of etoposide in the PEI/HD-PEI therapeutic group, cumulative doses of chemotherapy ultimately received by the patients (**Table 2**) corresponded to the recommended doses and were similar within a therapeutic group across age-groups.

**Table 2:** Median cumulative doses by therapeutic and age groups.

	Children (0-11 y) n=105		Adolescents (12-17 y) n=121		Adults (≥18 y) n=70		All n=296	
CarboPEI	65	62%	75	62%	55	79%	195	66%
Carboplatin mg/m² (min; max)	1200 (1000; 1200)		1200 (600; 2400)		1200 (600; 2400)		1200 (600; 2400)	
Ifosfamide mg/m² (min; max)	18000 (9000; 19000)		18000 (8500; 18300)		18000 (8500; 18000)		18000 (8500; 19000)	
Etoposide/Etoposide phosphate mg/m² (min; max)	1200 (1100; 1300)		1200 (300; 1600)		1200 (600; 1300)		1200 (300; 1600)	
PEI	32	31%	34	28%	11	16%	77	26%
Cisplatin mg/m² (min; max)	400 (100; 400)		400 (300; 420)		400 (320; 400)		400 (100; 420)	
Ifosfamide mg/m² (min; max)	30000 (460; 30500)		30000 (15000; 30500)		30000 (24000; 31000)		30000 (460; 31000)	
Etoposide/Etoposide phosphate mg/m² (min; max)	1200 (340; 1400)		1200 (900; 1300)		1200 (1100; 1300)		1200 (400; 1400)	
PEI/HD-PEI	8 (a)	8%	12 (b)	10%	4 (c)	6%	24	8%
Cisplatin mg/m² (min; max)	400 (300; 400)		400 (280; 400)		400 (330; 400)		400 (280; 400)	
Ifosfamide mg/m² (min; max)	33700 (17000; 38000)		34600 (24100; 35300)		32500 (28700; 34900)		33500 (17000; 38000)	
Etoposide/ Etoposide phosphate mg/m² (min; max)	3600 (2400; 3800)		3500 (2000; 4100)		2700 (2400; 3600)		3600 (2000; 4100)	

(a) 5 patients had 2 PEI + 2 HD-PEI, 2 patients had 3 PEI + 1 HD-PEI, 1 patient had 3 PEI + 2 HD-PEI; (b) 8 patients had 2 PEI + 2 HD-PEI, 4 patients had 3 PEI + 1 HD-PEI; (c) 2 patients had 2 PEI + 2 HD-PEI, 2 patients had 3 PEI + 1 HD-PEI. HD-PEI: haute dose PEI.

The vast majority of patients (n=289; 98%) received the four planned courses of treatment. Five patients (all with NGGCT) received only one (n=1 patient), two (n=2) or three (n=2) courses of chemotherapy (reason unknown). Conversely, two patients received five courses: one with HR-NGGCT received three PEI and two HD-PEI, and one patient with germinoma received a reduced dose course of etoposide-carboplatin and then two standard dose CarboPEI (reason unknown).

### Adverse events

The numbers of patients with at least one grade ≥3 haematological and/or non-haematological AE are presented by age-group (**Table 3**). In the whole population, haematological grade ≥3 toxicity was observed in a vast majority of patients (91%). Haematological grade ≥3 toxicity was less frequent in the adult group (77%) than in the child and adolescent groups (94 and 97%, respectively). Non-haematological grade ≥3 AE were rarer and mainly gastrointestinal or infectious, being observed in approximately one-third (31%) of patients. Again, the frequency was lower in the adult group (21%) than in the child and adolescent groups (31 and 36% respectively). When considering the occurrence of AE by therapeutic group, all patients with the PEI/HD-PEI strategy had at least one grade ≥3 AE. The rate of patients with at least one haematological grade ≥3 AE was slightly greater in patients receiving PEI/HD-PEI than in patients with PEI or CarboPEI strategies (100% vs. 92% and 90%, respectively). Non-haematological grade ≥3 AE were more frequent in PEI/HD-PEI and PEI strategies than in the CarboPEI strategy (79% and 42% vs. 21%, respectively) and were mainly gastrointestinal and infection/fever. In the PEI/HD-PEI group, three of 24 patients (12%) had grade ≥3 peripheral neurotoxicity, and two patients had grade ≥3 renal toxicity.

**Table 3:** Patients with at least one ≥ grade 3 adverse event according to age groups.

	Children (0-11 y) n=105		Adolescents (12-17 y) n=121		Adults (≥18 y) n=70		All n=296	
Hematological toxicities (%)	99	94%	118	98%	54	77%	271	92%
Non-hematological toxicities (%)	33	31%	44	36%	15	21%	92	31%
Digestive toxicity (%)	17	16%	29	24%	5	7%	51	17%
Infection/Fever (%)	17	16%	20	17%	7	10%	44	15%
Peripheral neurotoxicity (%)	6	6%	3	3%	2	3%	11	4%
Renal toxicity (%)	4	4%	3	3%	2	3%	9	3%
Auditory toxicity (%)	1	1%	2	2%	1	1%	4	1%

Multivariable analyses (**Table 4**) revealed that the occurrence of grade ≥3 haematological and non-haematological AE was neither statistically related to therapeutic group (p=0.9636), nor to sex (p=0.1617) but instead to age-group (p= 0.0011), with a lower risk for adults [OR=0.1 (0.03-0.4), when compared with adolescents. This was confirmed when considering only the subgroup of patients treated with CarboPEI (main group): adults treated with CarboPEI were less likely (p=0.0002) to experience a grade ≥3 AE [OR=0.1 (0.02-0.4)] when compared with adolescents treated similarly.

**Table 4:** Impact of age, sex and therapeutic group on the occurrence of grade ≥3 adverse event in the whole population and in the CarboPEI group.

	Occurrence of grade ≥3 adverse event				All; n=296		Odd ratio [95% confidence interval]	p-value
	No; n=24		Yes; n=272					
Age group								
Children (0-11 y)	6	25%	99	36%	105	35%	0.3 [0.1-1.4]	p:0.0011
Adolescent (12-17 y)	3	12%	118	43%	121	41%	Ref	
Adults (≥18 y)	15	63%	55	20%	70	24%	0.1 [0.03-0.4]	
Therapeutic group								
PEI	6	25%	71	26%	77	26%	0.9 [0.3-2.4]	p:0.9636
PEI/HD-PEI	0	0%	24	9%	24	8%	∞ [∞-∞]	
CarboPEI	18	75%	177	65%	195	66%	Ref	
Sex								
Male	23	96%	215	79%	238	80%	Ref	p:0.1617
Female	1	4%	57	21%	58	20%	4.5 [0.5-37.3]	

	Occurrence of grade ≥3 adverse event				Carbo PEI; n=195		Odd ratio [95% confidence interval]	p-value
	No; n=18		Yes; n=177					
Age group								
Children (0-11 y)	2	11%	63	36%	65	33%	0.9 [0.1-6.3]	p:0.0002
Adolescent (12-17 y)	2	11%	73	41%	75	39%	Ref	
Adults (≥18 y)	14	78%	41	23%	55	28%	0.1 [0.02-0.4]	

### Delayed chemotherapy

The interval between two courses of chemotherapy was similar in the three age-groups, with a median interval of 22 days (range 15-79 days). Twenty-nine percent of patients experienced delayed chemotherapy. This rate was higher in adolescents than in the paediatric or adult population (38% vs. 27% and 19%, respectively) (**Table 5**).

**Table 5:** Patients with at least one delayed course of chemotherapy according to age group.

	Children (0-11 y) n=105		Adolescents (12-17 y) n=121		Adults (≥18 y) n=70		All n=296	
Chemotherapy delayed 7 days or more	28	27%	46	38%	13	19%	87	29%
Reason for delayed chemotherapy								
Prolonged aplasia (%)	15	14%	27	22%	5	7%	47	16%
Renal toxicity (%)	0	0%	2	2%	0	0%	2	1%
Clinical status (%)	5	5%	5	4%	0	0%	10	3%
Other (%)	12	11%	15	12%	10	14%	37	13%

Logistic regression analyses (**Table 6**) revealed that the occurrence of delayed chemotherapy was not statistically related to sex (p=0.1591) but statistically related to therapeutic group (p=0.0164), with a greater risk for the PEI strategy [OR: 3.4 (1.4-8.5)] and was statistically related to patient age (p=0.0001), with a lower risk for adults [OR: 0.2 (0.1-0.4)] when compared with adolescents. This was confirmed when considering only the subgroup of patients treated with CarboPEI (main group), again adults were less statistically (p=0.0002) likely to experience a delay in chemotherapy [OR: 0.2 (0.1-0.4)] when compared with adolescents. Qualitative analysis of missing data demonstrated a similar percentage of missing data per cure across age-group (date not shown).

**Table 6:** Impact of age, sex and therapeutic group on delayed chemotherapy on the whole population and in the CarboPEI group.

	Delayed chemotherapy				All; n=296			Odd ratio [95% confidence interval]	p-value
	No; n=209		Yes; n=87						
Age group									
Children (0-11 y)	77	37%	28	32%	105	35%		0.5 [0.2-1.1]	0.0003
Adolescent (12-17 y)	75	36%	46	53%	121	41%		Ref	
Adults (≥18 y)	57	27%	13	15%	70	24%		0.2 [0.1-0.4]	
Therapeutic group									
PEI	57	27%	20	23%	77	26%		3.4 [1.4-8.5]	0.0164
PEI/HD-PEI	10	5%	14	16%	24	8%		3.0 [0.6-13.5]	
CarboPEI	142	68%	53	61%	195	66%		Ref	
Sex									
Male	165	79%	73	84%	238	80%		Ref	0.1591
Female	44	21%	14	16%	58	20%		2.0 [0.8-5.5]	

	Delayed chemotheraphy				Carbo PEI, n=195			Odd ratio [95% confidence interval]	p-value
	No; n=142		Yes; n=53						
Age group									
Children (0-11 y)	52	36%	13	24%	65		33%	0.6 [0.2-1.5]	0.0002
Adolescent (12-17 y)	45	32%	30	57%	75		39%	Ref	
Adults (≥18 y)	45	32%	10	19%	55		28%	0.2 [0.1-0.4]	

## Discussion

The current study investigated the impact of age on the occurrence of (CRAT). It unexpectedly did not reveal higher levels of CRAT in adults, but reported higher CRAT in the adolescent group. Apart from lower cumulative doses of etoposide in the small adult HR-NGGCT group, similar doses of chemotherapy were otherwise applied. These results seem not explained by under-reporting of AEs, although we cannot rule out the possibility that a longer interval between treatment and completion of the clinical formulary (CRF) in the adult department may have underestimated toxicity. This observation may rather reflect either an age-specific susceptibility to the drug but other explanations such as differences in the management of supportive care or at-risk behaviours could be considered.

The relationship between age and CRAT has been widely reported, but sometimes with conflicting data. Whereas in sarcoma it has been suggested that the toxicity was equivalent or inferior in the adult population (9–11) it seems the opposite for acute leukemia (12, 13). However, data regarding CRAT in the specific adolescent population is limited and underlines the heterogeneity of drug metabolism in this unique population (14). For example, it has been reported in medulloblastoma that alder patient (10-20 years old) may experience more CRAT than children aged 5-10 years when treated with identical chemotherapy (8). It is important to consider the adolescents as a separate age-group because of the specific physiology at this time: rapid growth and development. These changes may affect drug distribution, clearance, and the cytochrome P450 system (14, 15). Metabolism and clearance of most chemotherapy drugs are related to cytochrome P450 (CYP) isoenzymes, which play an important role in the biotransformation of anticancer agents. Activity of CYP enzymes has a wide inter-patient variation, but is also influenced by puberty, showing a decreased expression of CYP1A2, CYP2B6, and CYP2C19 as oestrogen and androgen levels rise (15, 16). Ifosfamide is an anticancer pro-drug metabolised and activated in the liver by the P450 cytochrome. In a series of 16 children aged 1 to 16 years, the estimated pharmacokinetic parameters (clearance, volume of distribution, and half-life) were dependent on body size and age but not any other patient variable (17). Clearance of ifosfamide thus appeared to be higher in paediatric patients compared with adolescents treated with the same dosing and mode of administration (17).

Several series have reported etoposide pharmacokinetics, but few have reported data regarding the relationship between etoposide disposition and age, and none were designed to specifically evaluate differences in clearance between young children and adolescents leading to conflicting data: some series describing an inverse correlation between clearance and age, whereas others didn’t (14). Clearance of cisplatin is influenced by renal function but also perfusion duration. A circadian variability of tubular uptake of cisplatin related to testosterone levels has also been reported (18). Whether this could explain heterogeneity within the adolescent population according to pubertal stage warrants further study.

In addition to physiological differences, adolescents may display more at-risk behaviours such as alcohol, tobacco, and/or drug use, unadvised dietary intake, or non-compliance with e.g., neutropenic hygiene rules. Compliance with supportive treatment (antibiotic, mucositis prophylaxis, etc.) might be less effective for adolescents, thus potentially leading to higher toxicity. Thus, adolescents have unique medical and psychosocial needs that warrant appropriate consideration and potential interventions (19).

Moreover, differences in CRAT incidence may represent different clinical approaches between paediatric and adult oncologists. Some studies reported that wider use of Granulocyte-Colony-Stimulating Factor (G-CSF) resulted in a lower incidence of neutropenic fever in adult patients (9, 10). Unfortunately, no data about the institution delivering treatment were available in the database and thus it is not possible to ascertain whether adolescents were treated in paediatric or adult units. We cannot exclude that a different use of G-CSF between paediatric and adult oncologists could have had some impact on the occurrence of haematological toxicity.

CNS-GCT are rarer in adults than in children/adolescents. In these situations of orphan disease, physicians responsible for such patients are encouraged either to share discussions in multidisciplinary boards (20) and/or to use existing paediatric protocols. Currently, international endeavours such as the European reference network EURACAN (https://euracan.eu/) encourage paediatric and adult neuro-oncology collaboration on orphan brain diseases. However, transposing a paediatric strategy to adult practice may be challenging. The SIOP-CNS-GCT-II European trial was opened without age limitation, offering the opportunity to include older patients than in the preceding SIOP-CNS-GCT-96 protocol. The current study encourages further trials without age limitations which has also recently become a reality for other brain tumours such as medulloblastoma (21). Such an approach will ensure the most equitable and representative trial enrollment and ensure improvements to patient outcomes will benefit all, regardless of age.

## Data availability statement

The raw data supporting the conclusions of this article will be made available by the authors, without undue reservation.

## Ethics statement

The studies involving humans were approved by the Comité de Protection des Personnes SUD-EST IV centre leon berard. The studies were conducted in accordance with the European legislation. Written informed consent for participation in the SIOP-CNS-GCTII trial was provided by the participants or participants' legal guardians.

## Author contributions

GP: Writing – review & editing, Writing – original draft, Conceptualization. CS: Writing – review & editing, Writing – original draft, Methodology, Investigation, Formal analysis. DF: Writing – review & editing, Writing – original draft, Visualization, Methodology, Conceptualization. AP: Writing – review & editing, Writing – original draft, Data curation. NG: Writing – review & editing, Writing – original draft, Data curation. R-DK: Writing – review & editing, Writing – original draft, Data curation. MP: Writing – review & editing, Writing – original draft, Visualization, Resources, Investigation. MZ: Writing – review & editing, Writing – original draft, Methodology, Data curation. MM: Writing – review & editing, Writing – original draft, Visualization, Validation, Data curation. JN: Writing – review & editing, Writing – original draft, Visualization, Validation, Data curation. GC: Writing – review & editing, Writing – original draft, Visualization, Validation, Investigation, Data curation, Conceptualization. CF-C: Writing – review & editing, Writing – original draft, Validation, Supervision, Project administration, Methodology, Investigation, Data curation, Conceptualization.
